# Where Does Blood Flow Restriction Fit in the Toolbox of Athletic Development? A Narrative Review of the Proposed Mechanisms and Potential Applications

**DOI:** 10.1007/s40279-023-01900-6

**Published:** 2023-08-14

**Authors:** Charlie J. Davids, Llion A. Roberts, Thomas Bjørnsen, Jonathan M. Peake, Jeff S. Coombes, Truls Raastad

**Affiliations:** 1 Sport, Performance, and Nutrition Research Group, School of Allied Health, Human Services and Sport, La Trobe University, Melbourne, Australia; 2 School of Human Movement and Nutrition Sciences, The University of Queensland, Brisbane, QLD Australia; 3 Sport Performance Innovation and Knowledge Excellence (SPIKE), Queensland Academy of Sport, Brisbane, QLD Australia; 4 School of Health Sciences and Social Work, Griffith University, Gold Coast, QLD Australia; 5 Department of Sport Science and Physical Education, University of Agder, Kristiansand, Norway; 6 Norwegian Olympic and Paralympic Committee and Confederation of Sports, Oslo, Norway; 7 School of Biomedical Science, Queensland University of Technology, Brisbane, QLD Australia; 8 Department of Physical Performance, Norwegian School of Sport Science, Oslo, Norway

## Abstract

Blood flow-restricted exercise is currently used as a low-intensity time-efficient approach to reap many of the benefits of typical high-intensity training. Evidence continues to lend support to the notion that even highly trained individuals, such as athletes, still benefit from this mode of training. Both resistance and endurance exercise may be combined with blood flow restriction to provide a spectrum of adaptations in skeletal muscle, spanning from myofibrillar to mitochondrial adjustments. Such diverse adaptations would benefit both muscular strength and endurance qualities concurrently, which are demanded in athletic performance, most notably in team sports. Moreover, recent work indicates that when traditional high-load resistance training is supplemented with low-load, blood flow-restricted exercise, either in the same session or as a separate training block in a periodised programme, a synergistic and complementary effect on training adaptations may occur. Transient reductions in mechanical loading of tissues afforded by low-load, blood flow-restricted exercise may also serve a purpose during de-loading, tapering or rehabilitation of musculoskeletal injury. This narrative review aims to expand on the current scientific and practical understanding of how blood flow restriction methods may be applied by coaches and practitioners to enhance current athletic development models.

## Key Points

**Table:** 

Both resistance and endurance exercise may be combined with blood flow restriction to provide a broad range of adaptations in skeletal muscle, benefitting both muscular strength and endurance qualities concurrently, which are necessary for athletic performance in most sports.
High-frequency blocks of blood flow restriction training may also selectively target satellite cells and promote myonuclear accretion, thereby priming skeletal muscle to attain greater hypertrophy in subsequent blocks of traditional high-load training.
Practitioners and coaches may also use blood flow restriction exercise in their periodisation models to supplement or de-load from traditional high-intensity training, or to accelerate recovery from musculoskeletal injury.
Further research is warranted to optimise these approaches in athletic cohorts and to evaluate their practical merit.

## Introduction

Performing exercise under partial blood flow restriction (BFR) has garnered much interest in recent years, owing to the potential to produce marked skeletal muscle hypertrophy and increases in strength with low training loads [1]. In particular, the magnitude of hypertrophic adaptations to resistance training (RT) is like that observed with traditional high-load RT (HL-RT), even when very low loads (< 30% of 1 repetition maximum [1RM]) are performed in conjunction with BFR [2]. Consequently, this method of exercise is recognised to have important implications for populations who cannot tolerate high levels of mechanical load, or would benefit from targeted periods of reduced mechanical stress. Such populations include the frail elderly [3] and those returning from musculoskeletal injury [4], but this also extends to athletic populations [5].

Evidence continues to emerge that athletes can use BFR to maximise various aspects of physical development, including both muscular strength and endurance capabilities [6–11]. Traditionally, it has been thought that these divergent phenotypes in skeletal muscle are dichotomous, and require different training protocols [12]. However, recent data suggest that both endurance and RT under BFR can elicit hypertrophic and oxidative capacity adaptations simultaneously [6, 7, 9]. This dual ability of BFR training makes it an attractive choice for many athletes because it can reduce the number of sessions required to maintain and develop such physical qualities. More recently, there has been a shift in the literature to understand whether the combination of low-intensity BFR in conjunction with traditional HL-RT provides a synergistic effect on training adaptations [6, 8, 13, 14]. These data provide another avenue through which coaches and their athletes may use BFR training to optimise athletic development.

Despite the literature supporting the use of BFR in athletes, evidence suggests that practitioners and coaches often limit its use to rehabilitation from musculoskeletal injury, both in athlete and non-athlete populations [15]. Presumably, this may be because of the limited scientific understanding on how healthy athletic cohorts should adopt this mode of exercise training, and how it should be prescribed in conjunction with more typical high-intensity training methods. Applications of BFR in general populations [16] and well-trained individuals and athletes [17] have been provided previously. However, some updated recommendations are warranted to reflect the significant amount of novel information now available, particularly with athletes.

In this narrative review, we evaluate the efficacy of BFR as an additional training method for athlete populations and outline several potential mechanisms of action. We also propose several novel strategies to inform practitioners on how to best use BFR exercise to supplement traditional high-load training to maximise athletic performance.

## Phenotypic Adaptations to Training

### Adaptations Towards a Strength and Power Phenotype

When BFR is combined with RT (BFR-RT), the principal adaptation is skeletal muscle hypertrophy. Studies consistently demonstrate increases in skeletal muscle size, both at the micro level (i.e. increases in myofibre area) [6, 18–20] and at the macro level (i.e. increases in muscle cross-sectional area [CSA]) [3, 6, 21–23] following BFR-RT, which are commensurate with HL-RT [2]. Importantly, this holds true even in well-trained and athletic cohorts [6, 24]. Muscular strength is also frequently reported to increase with BFR-RT [25–27]. Seemingly, strength adaptations to low-load (LL) RT can be enhanced with the addition of BFR [28]; however, these adaptations are often inferior to traditional HL-RT [2]. An in-depth discussion of the potential reasons for these divergent strength gains between LL-BFR and HL-RT is provided elsewhere [29, 30]. One possible reason for this discrepancy is sub-optimal neural adaptation following LL-BFR, which alongside hypertrophy, also mediates increases in strength [30]. It may be that the highest threshold motor units and muscle fibres are only briefly recruited during LL-BFR, particularly when sets are completed with a fixed repetition scheme (i.e. not to failure) [29]. This may also be compounded by the inhibitory influence of group III/IV afferent input to the central nervous system (CNS). Group III afferents, specifically, are sensitive to metabolic perturbations [31], and thus the activation of these afferents is related to the degree of ischaemia and the amount of active muscle mass during exercise [29]. Such afferent input may limit the firing rates of the highest threshold motor units. If the discharge rates of these units are limited consistently (by exclusively performing LL-BFR), this may limit maximal adaptation within the CNS. There is a paucity of literature comparing neural adaptations following LL-BFR and HL-RT [30]. However, some evidence in untrained individuals suggests that voluntary activation tends to increase with HL-RT, with negligible changes following LL-BFR [32, 33]. Hypothetically, such differences in neural adaptations and strength outcomes between protocols may be exacerbated in athletic cohorts, owing to elevated baseline levels of neuromuscular function. Other plausible explanations for the lower increases in strength with LL-BFR compared with HL-RT may be differences in structural and architectural adaptations, such as changes in tendon properties, muscle length and fascicle pennation angle in pennated muscles. There is, however, conflicting results on the impact on tendon properties [33–35] and information on possible differences in muscle length and architecture is lacking. Despite the inferior effect on muscle strength, it is important to note that muscular strength does not diminish, and still improves marginally following LL-BFR — even when adopted for prolonged periods in well-trained individuals [24]. Indeed, when HL-RT and LL-BFR are combined together in the same training programme (as we propose later in this review), slow-velocity strength adaptations may be superior compared with HL-RT alone [14, 36].

The influence of BFR on power-based qualities of the neuromuscular system is less certain. In theory, because power is the product of force and velocity, power may be enhanced somewhat with BFR through the development of strength. However, the velocity of muscle contraction is typically much slower with BFR-RT than traditional sprint or power training [37], which is likely due to: (i) the high degree of fatigue that is often generated by high repetition numbers and metabolite accumulation [38] and (ii) the intentional performance of repetitions at slower velocities and without maximal effort, seemingly to prolong time under tension. Furthermore, in contrast to HL-RT, adaptations to LL-BFR may favour slower-contracting, type I muscle fibres (discussed further below) [6, 39]. These notions are supported by results from a handful of studies that used jump or sprint performance as methods of assessing the impact of LL-BFR on lower body power. No improvements in vertical jump height [8, 40], horizontal jump distance [41] or sprint speed [8] were observed in these studies. Therefore, BFR-RT performed in its typical format (with slow-velocity contractions) may not influence the capacity for high-velocity movement, which may in turn explain the lack of changes in muscular power reported in the literature. Nevertheless, some evidence suggests that parameters associated with muscular power (such as the rate of force development [RFD]) are improved, yet exhibit a delayed response following LL-BFR. Nielsen et al. [42] observed a 15–20% increase in the RFD that was only apparent 12 days after the final LL-BFR training session. It is possible that previous studies that demonstrated negligible changes in muscular power may have evaluated these qualities too soon following training cessation and did not capture any delayed responses in these high-velocity movement qualities.

Moreover, muscular power may also be improved when BFR is applied in a less-conventional format. For example, the combination of BFR during sprint training enhanced RFD during an isometric leg press task and improved sprint time in a 100-m effort [43]. In addition, muscle thickness, used as a proxy indicator of muscle hypertrophy, simultaneously increased in the BFR condition [43]. Similarly, maximal power during a countermovement jump, and sprint time were enhanced in elite rugby players when BFR was applied intermittently (during working periods only) with HL-RT (70% 1RM) [27]. When BFR was applied during high-velocity knee extension training (300°/s), peak torque produced during a high-velocity maximal voluntary contraction increased beyond the same training without BFR [44]. Acutely, BFR may also be used with low-volume, high-load resistance exercise (HL-RE) to elicit a post-activation performance enhancement [45, 46]. Improvements in bench press bar velocity and power have been observed following a high-load BFR protocol that consisted of three repetitions at 70% 1RM [46]. Together, these data suggest that conventional LL-BFR has a negligible influence on muscular power; however, additional benefits (both acute and chronic) may be derived from combining BFR with high-intensity or high-velocity anaerobic training.

### Adaptations Towards an Endurance Phenotype

Typically, the specificity of the morphological and functional adaptations obtained by skeletal muscle depend on the implemented training modality [47]. Resistance training upregulates anabolic processes that ultimately translate to the chronic outcomes of muscle hypertrophy and strength gain [48]. Conversely, endurance training upregulates genes responsible for mitochondrial biogenesis and angiogenesis, which serves to improve metabolic functioning and enhance oxidative capacity and aerobic performance [49]. Interestingly, when BFR is combined with low-intensity endurance exercise, it appears that both hypertrophic and oxidative muscular adaptations can be simultaneously achieved. Conceição et al. [7] demonstrated that the combination of BFR with low-intensity cycling training can promote concurrent improvements in muscle CSA, strength and $\dot{V}$O_2max_. Of interest, the gains in CSA following BFR cycling were comparable to that observed with a traditional HL-RT condition, which involved leg press exercise. Furthermore, $\dot{V}$O_2max_ improved in the BFR cycling condition, despite the reduction in training intensity (40% $\dot{V}$ O_2max_) compared with the traditional endurance group (70% $\dot{V}$O_2max_). Another study involving well-trained cyclists reported that applying BFR in the recovery periods during sprint interval training significantly improved $\dot{V}$O_2max_ (~ 5%) above the same training without BFR [50]. However, this did not translate to improved cycling time-trial performance.

In contrast to the effect of traditional non-BFR training, both endurance and RT with BFR appear to influence local muscular endurance profoundly [8, 26, 51, 52]. Often, this is demonstrated by increased work, for example, volume or kilojoues completed before muscular failure occurs [8, 26], which is likely driven by the various mitochondrial and angiogenic mechanisms described below. The ability to offset fatigue-related impairments in performance is a valuable quality that transfers to a diverse range of sports. Crucially, improvements in local muscular endurance can occur simultaneously with muscle hypertrophy and muscular strength using BFR, which have traditionally been thought to be opposing adaptations in skeletal muscle [12].

## Postulated Mechanisms of Action

### Anabolic Signalling and Myofibrillar Protein Synthesis

The primary cellular mechanism through which LL-BFR purportedly enhances protein synthesis rates involves the mammalian target of rapamycin (mTOR) pathway [53]. The mTOR complex 1 acts as a key intracellular signalling protein that activates several downstream anabolic effectors [54]. The importance of this protein in the hypertrophic response to LL-BFR is demonstrated by the impaired muscle protein synthesis responses to exercise when rapamycin, a pharmaceutical inhibitor of mTOR, is administered [55]. Moreover, we have demonstrated that the upstream activation of the mTOR pathway is similar between LL-BFR and HL-RT [24], despite the differing contributions of mechanical and metabolic stimuli. In this instance, phosphorylation of several key protein kinases within both the mTOR and extracellular signal-regulated kinase pathways were similar following LL-BFR and HL-RT, and this translated into comparable hypertrophy following 9 weeks of training.

### Ribosomal Biogenesis

Alongside increases in muscle protein synthesis, another acute molecular response that occurs following a bout of resistance exercise (RE) is ribosomal biogenesis [56]. In the hours following exercise, increased transcription of ribosomal DNA occurs, and this is responsible for the accumulation of ribosomal RNA (rRNA), which is believed to precede hypertrophy [57]. Increases in ribosomal content would permit greater translational capacity, meaning the manufacture of contractile proteins is augmented. This is supported by evidence that RNA synthesis correlates strongly with muscle protein synthesis [56, 58, 59], with > 85% of total RNA being rRNA [60].

Recently, studies have investigated the influence of LL-BFR on rRNA responses. For example, we showed that LL-BFR acutely increases the expression of early rRNA transcriptional factors, such as c-Myc, transcription initiation factor IA and TATA box-binding protein-associated factor 1A, to a similar degree as HL-RT [24]. Further, Sieljacks et al. [59] demonstrated that LL-BFR increased cumulative RNA synthesis and total RNA content, again similar to HL-RT, after 6 weeks of training. The comparable responses between LL-BFR and HL-RT in stimulating ribosomal biogenesis may be driven through their similar activation of mTOR [24], which may be partly responsible for increases in rRNA [61]. This notion comes from evidence that inhibiting mTOR by rapamycin also blunts the increase in total RNA content and ultimately hypertrophy [62]. Together, these data suggest that both LL-BFR and HL-RT stimulate ribosomal biogenesis and that this process constitutes one underlying factor determining the muscle hypertrophic response to these two exercise protocols.

### Satellite Cell and Myonuclear Responses

Another mechanism through which BFR has been proposed to drive skeletal muscle hypertrophy is through activation of myogenic stem cells, known as satellite cells (SCs) [19]. Under certain conditions, SCs are activated to facilitate muscle repair and regeneration, as well as to provide new myonuclei during hypertrophy. Interestingly, SC proliferation is especially pronounced following high-frequency LL-BFR [18, 19], despite reduced loading conditions and often in the absence of typical biomarkers of muscle damage (e.g. creatine kinase) [63]. This suggests that BFR exercise may induce sufficient (and potentially excessive) myocellular stress, without overt structural damage. Several high-frequency BFR training studies (five or more sessions per week) support this notion; showing large increases in SC proliferation, alongside prolonged impairments in muscle function [18, 42, 64] and other markers of cellular stress, such as heat shock protein accumulation [63]. Interestingly, heat shock protein accumulation tended to occur at the myofibre membrane with LL-BFR, as opposed to a more granular myofibrillar staining pattern, which is more common following muscle damaging exercise [65].

Activating the SC pool adds myonuclei to existing muscle fibres, which supports the maintenance of a fixed cytoplasmic volume-to-nucleus ratio (also termed the *myonuclear domain*). A key rationale for such training-induced myonuclear addition is to provide more rDNA template to facilitate ribosome biogenesis (discussed above), which may be required to support the increased cytoplasmic volume of the growing myofibre [61]. Therefore, the addition of myonuclei would support rRNA transcription and diffusion throughout the myofibre, ultimately amplifying the hypertrophic response to RT through increased translational machinery. Importantly, although SC activation seems to be a transient process that may return to baseline levels quickly following 10–14 days of LL-BFR training [19], the newly acquired myonuclei seem to be maintained. Therefore, LL-BFR may serve as a strategy to target myonuclear accretion (and thus increased transcriptional capacity) through SC activation, when performed in 1- to 2-week blocks interspersed with traditional HL-RT [6]. In support of this notion, Nielsen et al. [19] and Bjørnsen et al. [6, 18, 64] have reported increases in myogenic SC numbers that range from ~ 100 to 200%, and increases in myonuclei content of ~ 20 to 30%, following ~ 20 days of high-frequency BFR training (five or more sessions per week). Such a robust increase in myonuclei content vastly exceeds the typical gains of ~ 9% during a longer duration (i.e. 16 weeks), traditional RT regime [66]. It is, however, important to acknowledge that the high-frequency protocols used in these LL-BFR studies generate substantial cellular stress [67] and prolonged decrements in muscle function [18, 42, 64], and therefore must be gradually introduced and used with caution. For example, in the study by Bjørnsen et al. [18], muscular strength was impaired (− 4%) 4 days following the high-frequency BFR block and only peaked (+ 6%) after 20 days of detraining.

The mechanism responsible for the potent activation of the SC pool by BFR exercise is not clear. However, one possible mechanism involves nitric oxide-induced synthesis of hepatocyte growth factor [53]. Nitric oxide synthase is linked to both mechanical stretch and shear stress [68], and increases acutely in muscle following LL-BFR [69]. Downstream of nitric oxide synthase, matrix metalloproteinases may mediate hepatocyte growth factor release from the extracellular matrix [68]. Once released, hepatocyte growth factor binds to its c-Met receptor, and consequently activates quiescent SCs in a dose-dependent manner [70]. Activation of the SC pool may also result from the downregulation of myostatin [71]. It has been reported that LL-BFR reduces myostatin gene expression [72], potentially by downregulating the messenger RNA (mRNA) expression of myostatin receptor activin IIb [73]. Myostatin is known to inhibit SC proliferation; accordingly, its downregulation may contribute to the robust activation of SCs described above [72].

### Mitochondrial and Angiogenic Responses

As discussed earlier, both resistance and endurance exercise combined with BFR elicit improvements in oxidative capacity [7, 50, 74] and local muscular endurance [8, 26, 51, 52]. Typically, such improvements are achieved in part by activating peroxisome proliferator-activated receptor gamma coactivator 1-α (PGC-1α), which is known as a master regulator of mitochondrial biogenesis [75]. Genes related to angiogenesis, like vascular endothelial growth factor, are also upregulated to support the expansion of new vasculature (enhanced capillarisation) and the development of existing blood vessels [76]. Whereas traditional endurance training upregulates both of these adaptive pathways, Conceição et al. [77] found that 15 min of BFR cycling did not increase mRNA expression of any of the isoforms of PGC-1α. However, in a subsequent study, the same authors found that 30 min of BFR cycling increased mRNA expression of cytochrome c subunit 4 isoform 1 [7], which is another marker indicative of mitochondrial biogenesis. Furthermore, a study by Christiansen et al. [78] observed increases in PGC-1α mRNA abundance following exercise when BFR was combined with interval running. The higher intensity of exercise used in the study by Christiansen et al. (105% of lactate threshold) may explain the greater effect on PGC-1α, which has been shown to respond in an intensity-dependent manner [79]. Notably, mitochondrial biogenesis is initiated by the translocation of PGC-1α from the cytosolic to the mitochondrial and nuclear compartment [49]. This activation of PGC-1α has been less studied. Additionally, more frequent cycling of hypoxia reperfusion is thought to enhance PGC-1α expression [80] and may also explain the discrepancies between studies. With regard to RT, it was recently observed that following both LL-BFR and traditional HL-RT, mitochondrial protein synthesis is elevated cumulatively, while mitochondrial respiratory function is also enhanced in untrained individuals [81]. Despite the similar mitochondrial adaptations between conditions, performance in a muscular endurance task was enhanced only in the group that trained with LL-BFR [81]. Differences in vascular adaptations induced by angiogenesis may have accounted for the disparity in endurance performance; however, capillary density was not assessed in this study. Interestingly, in strength-trained men and women, we have recently observed that levels of mitochondrial enzymes (e.g. citrate synthase, COX4) increase by 15–50% after 9 weeks of LL-BFR, whereas no change (or even a reduction) was observed in the same mitochondrial enzymes following HL-RT [82].

Previous studies have identified a robust increase in genes related to angiogenesis following acute RE with BFR [7, 69, 83, 84]. Hypoxic-inducible factor-1 is one such gene that is expressed following exposure to low-oxygen environments. Downstream targets of hypoxic-inducible factor-1 include vascular endothelial growth factor, which serves to enhance tissue function during low oxygen availability [76]. Vascular endothelial growth factor gene expression is routinely upregulated following both resistance and endurance exercise when combined with BFR [69, 76, 85], which can translate into enhanced capillarisation, in as little as 2 weeks [86]. In support of this notion, capillaries around type I fibres increased with LL-BFR training in a group of highly trained individuals, and this increase tended to be greater compared with those athletes who performed HL-RT only [6]. Another study (albeit in untrained individuals) observed a 15–16% increase in the capillary-to-muscle area ratio and thickening of the perivascular basal membrane following only 3 weeks of LL-BFR [87]. In the study described above by Conceição et al. [7], improvements in aerobic capacity were strongly associated with increases in angiogenic factors, as opposed to typical markers of mitochondrial biogenesis. Together, these findings suggest both endurance and RE combined with BFR can induce mitochondrial and angiogenic adaptations in skeletal muscle. In turn, these adaptations may be responsible for the improved aerobic capacity and muscular endurance performance that is often reported following training with BFR.

### Fibre-Type Specificity

A contentious issue in the current literature is the notion that BFR exercise may be used to target either type I or type II fibres preferentially. Few studies have employed the invasive biopsy techniques necessary to distinguish between fibre types, and to evaluate recruitment patterns, acute metabolism and chronic structural adaptations. Early research has clearly demonstrated that during LL-BFR, the full spectrum of fibre types is recruited to maintain force output—providing that sufficient volume is performed and the degree of fatigue is close to maximal [38, 88, 89]. Evidence of this is provided by the significant metabolite depletion (e.g. phosphocreatine, glycogen) in the larger type II fibres following BFR-RT, despite the use of low loads [39, 90]. However, more recent evidence suggests type I fibres may be subjected to greater stress with LL-BFR [6, 18, 39, 67, 91]. Specifically, this evidence stems from reports of greater heat shock protein responses and glycogen depletion in type I fibres following LL-BFR—even when sets are carried out until muscular failure [39, 67]. Wernbom and Aagaard [29] have suggested that motor unit recruitment patterns during LL-BFR may deviate from the Henneman size principle. Specifically, LL-BFR may preferentially utilise type I muscle fibres, which have a greater ability to tolerate low-oxygen conditions. The superior recovery capacity of type I fibres may also favour their continued recruitment across multiple sets of LL-BFR following the fatigue (and potential dropout of type II fibres) [29]. The recruitment pattern outlined above may explain how type I fibres are exposed to a greater stimulus following multiple sets of BFR exercise [29].

Factors such as training status and exercise selection may also play a role in determining which fibre types are exposed to the most stress, and subsequently adapt the most. In untrained populations, the relative increase in muscle fibre area following training appears to be equal between type I and type II fibres [19]. However, in a cohort of elite powerlifters, well accustomed to strength training, muscle fibre area appeared to increase solely in type I fibres, and myonuclei addition and capillarisation were also restricted to type I fibres [6]. Such findings may suggest that the type II fibres of these trained individuals are already well developed with their normal HL training, and there is a minimal additional effect from the LL-BFR stimulus. The use of BFR with bilateral compound exercises (such as the front squat, which was used in the study with powerlifters) may also stimulate group III/IV afferents within the muscle to a greater extent compared with single-joint unilateral exercises [29]. As discussed above, afferent input to the CNS may lead to submaximal recruitment and firing rates in high-threshold motor neurons that innervate type II fibres [29]. Taken together, this may explain why preferential adaptation was observed in type I fibres when a cohort of highly trained individuals performed bilateral compound exercises with BFR [6]. However, in untrained populations, and/or when performing unilateral single-joint exercises with BFR, there may be a more balanced stress application, and subsequent adaptation between type I and type II fibres. Nevertheless, the partitioning of stress across fibre types with BFR appears to be in contrast to traditional HL-RT, following which preferential type II fibre adaptation usually occurs in both untrained and trained individuals [48]. Together, these data suggest that LL-BFR can promote adaptations across both fibre types; however, there may be greater stress and subsequent adaptation in type I fibres, when compared with conventional HL-RT. Thus, LL-BFR in combination with conventional strength training may be of importance to optimise adaptation of both fibre types, and enhance functional outcomes such as strength and muscular endurance in highly strength-trained individuals.

## Athletic Applications

From the documented physiological effects reviewed in the first part, it is clear that even for athletes, who can tolerate high loads and intensities, BFR with low loads or intensities may also prove to be a valuable tool. Using this novel mode of exercise in conjunction with traditional training methods may provide a means to optimise physical development. Combining mechanical and metabolic stimuli could potentially elicit superior training adaptations (including both myofibrillar and oxidative capacity adaptations), compared with conventional training practices alone. However, if prescribed inappropriately, BFR exercise could significantly impair neuromuscular performance by generating considerable fatigue [18, 42], myocellular stress [63, 67] and in some cases, severe muscle damage [92, 93]. Thus, we feel it is important to provide practitioners and coaches with evidence-based recommendations for how to best use this mode of exercise with their athletes (Table 1). Potential avenues whereby BFR can be applied to athletic cohorts are discussed forthwith.

**Table 1:** Evidence-based recommendations for the use of BFR training for performance enhancement and injury management in a range of different sports and athletic populations

Desired outcome	BFR applications	Type of sport/athlete
*Improve muscular development* Increase muscle hypertrophy [6, 25, 26, 41] Increase muscular strength [41, 42, 103] Increase satellite cell proliferation [18, 19]	Supplemental BFR training (resistance training)	Team sports (e.g. soccer, netball, rugby) Racquet sports (e.g. tennis, squash) Centimetre, gram, second sports (e.g. powerlifting, track and field, swimming) Combat sports (e.g. mixed martial arts, boxing)^a^
	Sport-specific BFR training	
	High-frequency BFR block during off-season or pre-season	
*Improve local muscular endurance* Increase buffering capacity [8, 26, 97, 98] Increase capillary formation [87] Increase artery diameter [52, 141] Increase mitochondrial content [81, 82]	Supplemental BFR training (resistance training)	Team sports (e.g. soccer, netball, rugby) Racquet sports (e.g. tennis, squash) Endurance sports (e.g. triathlon, long course cycling) Centimetre, gram, second sports (e.g. powerlifting, track and field, swimming) Combat sports (e.g. mixed martial arts, boxing)
	Supplemental BFR training (endurance training)	
	Sport-specific BFR training	
	High-frequency BFR block during off-season or pre-season	
*Improve aerobic capacity* Increase maximal oxygen uptake [7, 142] Increase running economy [97]	Supplemental BFR training (endurance training)	Team sports (e.g. soccer, netball, rugby) Racquet sports (e.g. tennis, squash) Endurance sports (e.g. triathlon, long course cycling) Combat sports (e.g. mixed martial arts, boxing)
	Sprint interval training with BFR during recovery periods	
	Sport-specific BFR training	
*Support musculoskeletal rehabilitation* Limit muscle atrophy [114, 124, 126, 129] Limit strength loss [115, 126, 129] Limit reduction in oxidative capacity [74, 125] Pain reduction [131, 132] Potential fracture healing [116, 118–120]	Cyclical BFR during passive rest or NMES	Team sports (e.g. soccer, netball, rugby) Racquet sports (e.g. tennis, squash) Endurance sports (e.g. triathlon, long course cycling) Centimetre, gram, second sports (e.g. powerlifting, track and field, swimming) Combat sports (e.g. mixed martial arts, boxing)
	BFR applied during walking or low-intensity cycling	
	BFR combined with bodyweight exercises and light resistance	
	Supplemental BFR training (resistance training)	
*Improve performance in sport-specific tasks* Improved sprint performance [27, 41, 140] Improved change of direction [140]	Supplemental BFR training (resistance training)	Team sports (e.g. soccer, netball, rugby) Racquet sports (e.g. tennis, squash)
	High frequency BFR blocks	
	Sport-specific BFR training^b^	
*Training load management* Improvements in hypertrophy, strength, power, and endurance with reduced volume [111] Concurrent muscular and mitochondrial adaptations from single session [7, 82]	Supplemental BFR training (resistance training)	Endurance sports (e.g. triathlon, long course cycling) Centimetre, gram, second sports (e.g. powerlifting, track and field, swimming)
	Supplemental BFR training (endurance training)	
	High-frequency BFR blocks	
Post-activation performance enhancement in strength and power tasks [45, 46]	BFR during low-volume high-intensity priming exercise	Centimetre, gram, second sports (e.g. powerlifting, track and field, swimming)

Several BFR applications are presented for each sport/athlete. Desired outcomes are supported with evidence using relevant athletic populations, or well-trained individuals, where possible

*BFR* blood flow restriction, *NMES* neuromuscular electrical stimulation

^a^Hypertrophy may be undesirable in some combat sport athletes because of the use of weight classes. It is recommended that the use of BFR is carefully considered in these sports

^b^The metabolic demand is increased with BFR during sport-specific training, which may impair the movement quality and coaches should therefore carefully consider the possible benefits on physiological adaptations vs possible negative effects on the technical execution of drills

### Supplemental BFR Exercise

Low-load BFR training is well tolerated and efficacious in a diverse range of athletes, with improvements observed in several pertinent outcomes, including muscle CSA [6, 25, 41], strength [6, 14, 27], local muscular endurance [8, 26] and sport-specific performance tests [26, 27, 41]. Despite this, LL-BFR should not be seen as a replacement or surrogate for conventional training methods with high loads/intensities, which remain the gold standard training strategies for athletic development. Emerging evidence suggests that high loading conditions may not be required for optimal bone [94] and tendon responses [34, 35]; nevertheless, it appears that high loads do promote superior strength [2] and power development [40], potentially mediated through enhanced neural and structural adaptations. Indeed, muscular adaptations to LL-BFR training are enhanced when integrated with traditional HL-RT [95]. Consequently, there has been a shift towards the use of LL-BFR as an additional supplementary stimulus to traditional training practices.

#### Same-Session Supplementary BFR

Performing BFR exercise after completing regular high-load exercise within a single session enhances bench press [14] and back squat [14, 36] 1RM in well-trained American football players. Despite these strength improvements, hypertrophy (as inferred by girth measurements) only appeared to increase in the chest, with no differences in arm or thigh girth compared with a control group who completed LL training without BFR [14]. The use of such anthropometric measurements as proxy indicators of muscular hypertrophy in these studies is a limitation.

To address this, Scott et al. [8] used ultrasound to measure muscle thickness in a 5-week training study involving Australian rules football athletes. Despite positive changes in strength and muscular endurance qualities over the training period, supplemental BFR training (following the completion of HL-RT) did not appear to enhance these attributes above the same training without BFR. Moreover, there were no significant changes in either condition for muscle thickness, and performance during sprinting and jumping tasks. It is unclear why the training did not result in muscular hypertrophy, given the findings from other studies that BFR promotes hypertrophic adaptations even in well-trained participants [25, 26, 41]. The authors proposed a possible interference effect caused by concurrent endurance training, as field-based conditioning sessions were completed immediately following the RT sessions with BFR [8]. Therefore, if optimal hypertrophy is desired it may be critical to consider the timing of concurrent training in relation to BFR training, which also is the case for traditional HL-RT. The 5-week training period might also have been too short to induce more robust adaptations in these well-trained athletes. Together, these data suggest supplemental BFR exercise performed in the same session as HL-RE may augment strength beyond HL-RE alone. Hypertrophic responses to same-session supplemental BFR exercise are, however, less clear, and warrant further investigation using gold-standard imaging measurement techniques (e.g. magnetic resonance imaging) and longer training blocks (> 6 weeks).

#### Separate Session/Block BFR

Conversely, supplementary BFR training may be completed in separate sessions or blocks from traditional training. A common approach in the literature is a block of high-frequency (five or more sessions/week) BFR training interspersed with traditional high-load training. As with same-session supplementary BFR exercise, separate sessions or blocks where LL-BFR exercise is exclusively performed could theoretically be used amongst HL-RT to provide a well-rounded stimulus for skeletal muscle (comprising metabolic and mechanical components). Moreover, the maintenance of an adaptive stimulus, despite shorter session durations, and lower mechanical load and volume may provide benefit through the management of the training load.

Supplemental BFR in separate sessions or blocks has not been investigated in athletic cohorts. However, studies using recreationally active individuals may offer some guidance in this area. Yasuda et al. [95] reported that combining LL-BFR and HL-RT in separate sessions (2× LL-BFR, 1× HL-RT per week) provided superior strength gains to LL-BFR alone. However, muscle CSA and strength following the combined training were comparable to HL-RT alone. One limitation of this study was that only HL-RT progressed in load across the 6 weeks, whereas the load remained the same for LL-BFR. Thus, LL-BFR sessions may not have induced an optimal stimulus. More recently, one study explored alternating weekly blocks of LL-BFR and HL-RT for a 6-week period [13]. Similar to the findings of Yasuda et al., there was no additional benefit for muscle size or strength adaptations when LL-BFR blocks were included in the training, compared with HL-RT alone. Of interest, no elevations in SC content or increases in myonuclei number were observed with the group that alternated between LL-BFR and HL-RT, in contrast to other LL-BFR studies (which are discussed below). Together, these data suggest that including LL-BFR training in separate sessions or blocks alongside HL-RT does not compromise muscle strength or size adaptations (and may preferentially target type I fibres); however, the inclusion of LL-BFR training may not provide overall greater strength or size adaptations to HL-RT alone. Further study of integrated programs combining LL-BFR and HL-RT, particularly involving athletes, is warranted.

#### Sport-Specific BFR Training

A novel use of BFR that has emerged in recent years is its application during sport-specific training. A handful of studies have reported that BFR during soccer-specific or futsal-specific training, such as small-sided games and drills, may improve various physical attributes above and beyond the same training performed without BFR. The application of BFR during futsal small-sided games was observed to increase levels of anabolic hormones and isometric knee extension strength [96], and anaerobic power and running economy [97] more than the group that trained without BFR. The authors postulated that the improvements in anaerobic and aerobic performance were likely because of local muscular adaptations, as improvements in $\dot{V}$O_2max_ were not different between groups [97]. Additionally, there tended to be larger improvements in a futsal-specific performance test in the BFR group, with large effect sizes observed. However, this was not statistically significant. The lack of statistical significance in some physical variables may be because of the short training intervention of 3 weeks (ten sessions).

More recently, Hosseini Kakhak et al. [98] performed a longer training intervention lasting 6 weeks (18 sessions) in soccer athletes. Significantly greater improvements were noted in the tests of aerobic power, sport-specific endurance, change of direction and muscular endurance following training with BFR. Performance in strength and power tests also improved; however, these were not significantly different between groups. Taken together, these studies suggest that sport-specific training combined with BFR exacerbates the metabolic demands of the drills, thus facilitating improvements in the buffering capacity of skeletal muscle and fatigue resistance. These findings are particularly relevant for coaches and practitioners who are required to concurrently develop numerous physical attributes in team sport athletes, while also improving their technical and tactical skills.

### High-Frequency Blocks

When individuals are unaccustomed to LL-BFR, this mode of exercise can induce significant muscle damage [92, 93], and prolonged decrements in force that are similar to unaccustomed eccentric exercise [99]. Despite this, once individuals become accustomed to LL-BFR (through protective adaptations, such as the repeated bout effect [100]), most data tend to suggest that the risk of muscle damage is similar to conventional HL-RT [39, 63, 99]. Recovery of exercise-induced impairments in neuromuscular function also appears to be very rapid [101, 102]. This has led some authors to suggest that higher training frequencies may be tolerated, which in turn may accelerate and augment adaptations within skeletal muscle. Some studies have successfully implemented training frequencies as high as twice daily [20, 41, 103, 104]. Following training blocks of 1–2 weeks (during which 12–24 sessions were completed), hypertrophy and strength improvements are similar in magnitude to those observed following much longer (i.e. 8–12 weeks) traditional HL-RT programmes [18–21, 41, 42, 104]. Such an approach was applied to elite powerlifters in a recent study, where two 1-week blocks of high-frequency LL-BFR training (five sessions per block) were interspersed amongst more typical high-load (~ 85% 1RM) training [6]. Following the 7-week training period, the LL-BFR and HL-RT group demonstrated greater hypertrophy and increase in isokinetic strength than HL-RT only, which were both correlated with increases in the type I fibre area. These data promote the use of high-frequency BFR blocks as a strategy to target type I muscle fibres in strength-trained individuals, drive SC proliferation and enhance the myonuclear number.

As higher training frequencies with LL-BFR are seemingly well tolerated [6, 41], this approach may represent a potential strategy to amplify and accelerate muscular development in periods where there is an emphasis on adaptation, such as the pre-season. It should, however, be highlighted that blocks of high-frequency BFR generate substantial myocellular stress (even if muscle damage is not observed), and especially when athletes are not familiar with LL-BFR [6]. Often this can lead to a transient over-reaching state in which myofibre size, strength and RFD are impaired initially, before supercompensation occurs [18, 42, 64]. Such protocols should therefore be carefully introduced and gradually progressed over time, to ensure that protective adaptations (i.e. repeated bout effect) can take place to minimise the risk of inducing excessive muscle stress and/or damage. Following familiarisation, frequencies of 5–7 sessions per week may be suitable, gradually building to 3–5 sets of exercise. It is recommended that sets are not initially performed until muscular failure; however, this can be introduced once the individual becomes acclimated to the BFR stimulus. For example, initially the final set of an exercise could be completed until muscular failure, before eventually progressing to completing all sets of the exercise until failure. Importantly, it is recommended that such protocols are tested early in the pre-season and avoided close to the competitive season. Finally, it must be acknowledged that it is not always feasible to fit such a high frequency of BFR sessions in an athletic programme; particularly for team sports, which have multiple sporting and physiological targets during a training phase. However, as outlined earlier in this review, a broad range of distinct adaptations (strength, hypertrophy, muscular endurance, aerobic power) can be concurrently obtained with BFR training. Nevertheless, we advocate for a high frequency early in pre-season, when technical and tactical training volume is lower.

### Managing Training Stress, De-Loading and Tapering

A key concern in elite sport is appropriately periodising training, which requires systematic planning to balance the goals of adaptation, recovery and performance [105]. An important aspect of periodisation involves phases of reduced loading and/or volume to allow for the recovery of particular tissues in the body, and to prepare for competition and/or the increased demand of the next training phase [106]. Another potential avenue in which BFR may be applied to athletes is during periods of tapering or de-loading. As discussed above, the lowered intensities and exercise volume would temporarily minimise the strain on joints, tendons, ligaments and the CNS. The recovery of muscle tissue during these periods is also a priority, which brings into question whether this would be a true ‘taper’ phase if BFR was used during these periods. This has yet to be investigated, which makes it difficult to speculate on the matter. Through our practical work with athletes we have explored this approach in powerlifters, anecdotally observing positive outcomes in subjective ratings of performance and recovery from the athletes. As stated above, although LL-BFR exercise can generate substantial myocellular stress and muscle damage, these outcomes appear to be minimised once an individual becomes accustomed to the stimulus, and if muscular failure is avoided [63, 99, 107, 108]. We have observed that following acclimation to LL-BFR training, robust hypertrophy signalling and expression of ribosomal RNA transcription factors still occur without prolonged decrements in neuromuscular function or other signs of muscle damage [24]. Thus, it may be possible to continue to achieve muscular development through metabolic stimuli, while simultaneously allowing muscle and other tissues to recover from the high mechanical stresses typically imposed by traditional progressive HL-RT.

Another primary concern in elite sport is managing total training stress to maximise physical performance, while also avoiding overtraining and injury [105]. Many sports require the continuous development of many different physical attributes (e.g. strength, speed, power) in conjunction with more sport-specific skills training [105]. Such a requirement demands a high number of training sessions, which can be problematic to fit into a weekly training schedule, especially during the competitive season. The accumulation of these training loads can also be physically demanding on an athlete’s body [109]. A hallmark of LL-BFR is the reduction in volume load (especially the reduction in absolute load) that is required to stimulate muscular adaptation [110, 111]. As the capacity for skeletal muscle to recover during rest periods is compromised because of a lack of oxygen, fatigue occurs earlier and with lower repetition numbers [38]. Consequently, the exercising muscles can receive a robust stimulus for adaptation, despite a potential reduction in exercise volume. Such a reduction in exercise volume serves as an efficient strategy because it minimises both total training stress, and training duration. The unique ability of LL-BFR to elicit both hypertrophic and oxidative capacity adaptations from the same stimulus means that training frequency may also be reduced [7, 52, 82]. Furthermore, the limited rest periods associated with this style of training also make it more time efficient than HL-RT.

### Accelerating Return to Play Following Musculoskeletal Injury

**Fig. 1:**
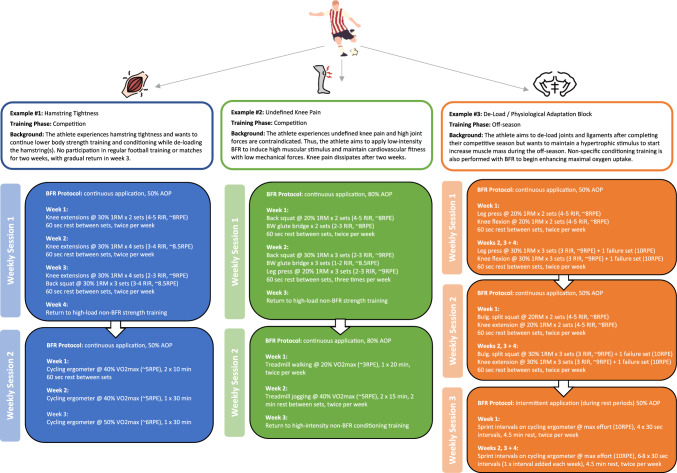
Three exemplary cases of how blood flow restriction (BFR) may be introduced and progressed for a team sport athlete at various times throughout the season. The example programmes provided a focus on lower body strength and conditioning training as these are most relevant to the case study sport. Other training modes (e.g. upper body strength, sport-specific training) may continue as normal if appropriate. In situations where one repetition maximum (1RM) testing is not possible or practical, it is recommended to use a load that is estimated to be between 30 and 35 RM and to adjust effort using the repetitions in reserve (RIR) and rating of perceived exertion (RPE) values provided. If measurement of repetition velocity is possible, practitioners may also use loads that equate to initial mean concentric velocities of 1.0–1.3 m per second. *Higher cuff pressures are used in the knee pain case study as these appear to elicit a greater hypoalgesic effect [131]. *AOP* arterial occlusion pressure, *BW* bodyweight

Musculoskeletal injuries are an unavoidable consequence in elite sport, given the high training loads, physical contact and high-impact forces imposed on the body [109]. Following musculoskeletal injuries, there is often a period of immobilisation or reduced loading necessary to allow the injured tissue to recover. Such periods of reduced loading have a deleterious effect on muscle mass and function (e.g. strength, muscular endurance) [112]. Therefore, the goal during rehabilitation of a musculoskeletal injury is to mitigate and reverse such atrophy and detriments in muscle function, to accelerate a return to pre-injury conditions. However, traditional strength-based and field-based training are contraindicated early in the rehabilitation process, owing to the high levels of mechanical tension and impact forces imposed on injured tissues [4]. Blood flow restriction, both at rest and when combined with various modes of muscle contraction, is a promising avenue to commence the rehabilitation process earlier, and offset atrophy and loss of strength [113]. The ischaemic stimulus offered by BFR can preserve and promote adaptation in muscle, despite the reduced levels of mechanical stress that are imposed on the body. Ultimately, this would allow injured athletes to fast track their recovery to pre-injury conditions, and accelerate their return to play (Fig. 1).

A four-step model has been proposed by Loenneke et al. [113], which outlines how BFR could be used in the gradual progression from (i) bed rest or immobilisation, (ii) walking or low-intensity cycling exercise, (iii) LL-RE and finally, (iv) HL-RE. The first stage of this progressive model has been derived from evidence suggesting that BFR alone (without exercise) can reduce the amount of muscle atrophy [114] and strength loss [115] that occurs with periods of unloading. There is also some evidence that intermittent periods of BFR may promote fracture healing [116–120]. A recent and novel addition to this early phase of rehabilitation is combining BFR with neuromuscular electrical stimulation (NMES) to induce involuntary muscle contraction. Alone, this technique appears to generate a modest degree of mechanical tension [121]; however, this is amplified under the ischaemic conditions [122, 123]. Acute studies have reported robust increases in lactate accumulation and growth hormone secretion [123], and significant neuromuscular fatigue following NMES and BFR, particularly when higher pressures are used [122]. The strength of the stimulus provided by NMES and BFR appears sufficient to inhibit muscle atrophy [124], and capillary regression [125] when repeated long term. Importantly, this stimulus can be achieved without joint movement or weight bearing, making it suitable for the initial stages of musculoskeletal rehabilitation. The use of isometric voluntary contractions may be used as a surrogate to NMES under circumstances where applying electrodes is not possible (e.g. orthopaedic casts), or access to NMES is limited.

Stage 2 of the model involves early engagement in very LL isolated activities (e.g. resistance bands) that are typical in rehabilitation programmes. The goals of such activities are to increase range of motion and develop strength, while avoiding aggravating the injured tissue. Applying BFR enhances the effectiveness of these exercises by increasing metabolic stress within the muscle [4]. In support of this notion, Ohta et al. [126] demonstrated that adding BFR to a typical anterior cruciate ligament rehabilitation programme mitigated strength losses, and improved the ratio of healthy-to-injured thigh CSA. Once weight bearing is tolerated, BFR may be employed with walking or similar low-intensity exercise (e.g. cycling). Unlike low-intensity aerobic exercise under unrestricted conditions (i.e. without BFR), BFR during walking and cycling elicits substantial muscle hypertrophy (~ 5% increase in CSA) [127, 128], increases in muscular strength (~ 5 to 10%) [128–130] and aerobic capacity (~ 10%) [74] in athletic cohorts. Importantly, these modes of exercise can be employed early in the rehabilitation process of most musculoskeletal injuries, given the very low levels of mechanical stress involved.

Following this, conventional REs can be implemented with BFR using low-to-moderate loads (20–40% 1RM). A systematic review and meta-analysis demonstrated that LL-BFR is superior to LL-RT in rehabilitating musculoskeletal conditions such as anterior cruciate ligament injury and osteoarthritis and in older adults at risk of sarcopenia [4]. Data are also emerging to suggest that LL-BFR provides an acute analgesic effect, which is not observed with LL-RE or HL-RE [131, 132]. Thus, LL-BFR may also improve tolerance and adherence to rehabilitation programmes.

The final step in the model is the return to traditional HL-RT, which is likely necessary for optimal strength, power, and neural and structural adaptations [4, 133]. Although equivocal, traditional HL-RT may also be required to restore the integrity of tendons and bones [34, 134], especially if recovering from injury to these tissues [135]. However, LL-BFR may still be used as a supplement in this phase to provide a well-rounded stimulus for muscle adaptation. Specifically, following periods of detraining it appears that oxidative fibres (e.g. type I and type IIa) are negatively affected more so in trained individuals [136, 137]. We also speculate that the pronounced SC proliferations in response to high-frequency BFR training (which has been discussed earlier in this review) may serve as a potential strategy to accelerate the regeneration of muscle-based injuries, such as muscle strains. Following SC proliferation, these cells give rise to myogenic precursor cells, known as myoblasts, which ultimately form new myotubes or fuse with damaged myofibres [48]. However, no studies have examined this possibility to date. Therefore, it may be beneficial to continue to perform LL-BFR training throughout the final stages of rehabilitation, which may specifically stress oxidative fibres and drive SC proliferation, leading to preferential hypertrophic and mitochondrial adaptations [6, 39] and myofibre regeneration [48].

### Safety Considerations

The safety of restricting blood flow during exercise has been rigorously investigated over the past two decades and has been reviewed extensively elsewhere [16]. Although several theoretical concerns about the safety of BFR exist, the resulting consensus is that exercise in combination with BFR poses no greater risk than regular exercise when evidence-based recommendations are followed [22, 138]. Potential complications to BFR exercise that have been proposed include vascular dysfunction, venous thromboembolism, nerve injury and elevated cardiac demand due to excessive stimulation of the muscle metaboreflex, oxidative stress and rhabdomyolysis. In healthy athlete cohorts, the risk of the above complications is thought to be extremely low [16, 139]. However, certain conditions may elevate the risk of these complications, such as peripheral vascular, cardiovascular or clotting disorders [22]. Thus, it is imperative that athletes are screened prior to commencing any BFR exercise programme using an appropriate tool [139]. As we have already alluded to earlier in this review, we recommend that BFR protocols are gradually introduced to obtain the repeated bout effect [99], and to minimise the risk of rhabdomyolysis.

## Conclusions

Blood flow-restricted RE is currently recognised as a LL time-efficient approach to reap many of the benefits of typical HL-RT. Consequently, this method of training holds great promise for populations who cannot tolerate the high levels of mechanical stress imposed by heavy external loads. As outlined in this review, many avenues exist in which BFR may also benefit athletic development (Fig. 1). Both resistance and endurance exercise may be combined with BFR to provide a spectrum of adaptations in skeletal muscle, spanning from myofibrillar to mitochondrial adaptations. Such diverse adaptations would benefit both muscular strength and endurance qualities concurrently, which are demanded in athletic performance, especially in some team sports. High-frequency blocks of BFR training may also selectively target the SC pool and promote myonuclear accretion, thereby priming skeletal muscle to attain greater hypertrophy in subsequent blocks of traditional HL-RT. Practitioners and coaches may also use BFR exercise in their periodisation models to de-load from traditional HL-RT, or to accelerate recovery from musculoskeletal injury. Despite the promise of the applications described, it is important that practitioners and athletes introduce the BFR stimulus carefully, and gradually, to attain protective adaptations, and avoid excessive muscle damage, cellular stress and prolonged decrements in neuromuscular function. In summary, there is a plethora of theoretical avenues in which training with BFR may be applied alongside traditional practices to maximise athletic development. These approaches need to be validated in future research studies comprising athletic cohorts in order to evaluate their practical merit.

## References

[1] Loenneke JP, Wilson JM, Marin PJ, Zourdos MC, Bemben MG (2012). Low intensity blood flow restriction training: a meta-analysis. Eur J Appl Physiol.

[2] Lixandrão ME, Ugrinowitsch C, Berton R, Vechin FC, Conceição MS, Damas F (2018). Magnitude of muscle strength and mass adaptations between high-load resistance training versus low-load resistance training associated with blood-flow restriction: a systematic review and meta-analysis. Sports Med.

[3] Vechin FC, Libardi CA, Conceicao MS, Damas FR, Lixandrão ME, Berton RP (2015). Comparisons between low-intensity resistance training with blood flow restriction and high-intensity resistance training on quadriceps muscle mass and strength in elderly. J Strength Cond Res.

[4] Hughes L, Paton B, Rosenblatt B, Gissane C, Patterson SD (2017). Blood flow restriction training in clinical musculoskeletal rehabilitation: a systematic review and meta-analysis. Br J Sports Med.

[5] Scott BR, Loenneke JP, Slattery KM, Dascombe BJ (2016). Blood flow restricted exercise for athletes: a review of available evidence. J Sci Med Sport.

[6] Bjørnsen T, Wernbom M, Kirketeig A, Paulsen G, Samnoy L, Baekken L (2019). Type 1 muscle fiber hypertrophy after blood flow-restricted training in powerlifters. Med Sci Sports Exerc.

[7] Conceição MS, Junior EMM, Telles GD, Libardi CA, Castro A, Andrade ALL (2019). Augmented anabolic responses following 8-weeks cycling with blood flow restriction. Med Sci Sports Exerc.

[8] Scott BR, Peiffer JJ, Goods PSR (2017). The effects of supplementary low-load blood flow restriction training on morphological and performance-based adaptations in team sport athletes. J Strength Cond Res.

[9] Vissing K, Groennebaek T, Wernbom M, Aagaard P, Raastad T (2020). Myocellular adaptations to low-load blood flow restricted resistance training. Exerc Sport Sci Rev.

[10] Wortman RJ, Brown SM, Savage-Elliott I, Finley ZJ, Mulcahey MK (2021). Blood flow restriction training for athletes: a systematic review. Am J Sports Med.

[11] Pignanelli C, Christiansen D, Burr JF (2021). Blood flow restriction training and the high-performance athlete: science to application. J Appl Physiol.

[12] Hughes DC, Ellefsen S, Baar K (2018). Adaptations to endurance and strength training. Cold Spring Harb Perspect Med.

[13] Hansen SK, Ratzer J, Nielsen JL, Suetta C, Karlsen A, Kvorning T (2020). Effects of alternating blood flow restricted training and heavy-load resistance training on myofiber morphology and mechanical muscle function. J Appl Physiol.

[14] Yamanaka T, Farley RS, Caputo JL (2012). Occlusion training increases muscular strength in division IA football players. J Strength Cond Res.

[15] Patterson SD, Brandner CR (2018). The role of blood flow restriction training for applied practitioners: a questionnaire-based survey. J Sports Sci.

[16] Patterson SD, Hughes L, Warmington S, Burr J, Scott BR, Owens J (2019). Blood flow restriction exercise: considerations of methodology, application, and safety. Front Physiol.

[17] Scott BR, Loenneke JP, Slattery KM, Dascombe BJ (2015). Exercise with blood flow restriction: an updated evidence-based approach for enhanced muscular development. Sports Med.

[18] Bjørnsen T, Wernbom M, Lovstad AT, Paulsen G, D'Souza RF, Cameron-Smith D (2018). Delayed myonuclear addition, myofiber hypertrophy and increases in strength with high-frequency low-load blood flow restricted training to volitional failure. J Appl Physiol.

[19] Nielsen JL, Aagaard P, Bech RD, Nygaard T, Hvid LG, Wernbom M (2012). Proliferation of myogenic stem cells in human skeletal muscle in response to low-load resistance training with blood flow restriction. J Physiol.

[20] Yasuda T, Abe T, Sato Y, Midorikawa T, Kearns CF, Inoue K (2005). Muscle fiber cross-sectional area is increased after two weeks of twice daily KAATSU-resistance training. Int J KAATSU Train Res.

[21] Abe T, Beekley MD, Hinata S, Koizumi K, Sato Y (2005). Day-to-day change in muscle strength and MRI-measured skeletal muscle size during 7 days KAATSU resistance training: a case study. Int J KAATSU Train Res.

[22] Clark BC, Manini TM, Hoffman RL, Williams PS, Guiler MK, Knutson MJ (2011). Relative safety of 4 weeks of blood flow-restricted resistance exercise in young, healthy adults. Scand J Med Sci Sports.

[23] Lixandrão ME, Ugrinowitsch C, Laurentino G, Libardi CA, Aihara AY, Cardoso FN (2015). Effects of exercise intensity and occlusion pressure after 12 weeks of resistance training with blood-flow restriction. Eur J Appl Physiol.

[24] Davids CJ, Næss TC, Moen M, Cumming KT, Horwath O, Psilander N (2021). Acute cellular and molecular responses and chronic adaptations to low-load blood flow restriction and high-load resistance exercise in trained individuals. J Appl Physiol.

[25] Takarada Y, Sato Y, Ishii N (2002). Effects of resistance exercise combined with vascular occlusion on muscle function in athletes. Eur J Appl Physiol.

[26] Manimmanakorn A, Manimmanakorn N, Taylor R, Draper N, Billaut F, Shearman JP (2013). Effects of resistance training combined with vascular occlusion or hypoxia on neuromuscular function in athletes. Eur J Appl Physiol.

[27] Cook CJ, Kilduff LP, Beaven CM (2014). Improving strength and power in trained athletes with 3 weeks of occlusion training. Int J Sports Physiol Perform.

[28] Slysz J, Stultz J, Burr JF (2016). The efficacy of blood flow restricted exercise: a systematic review & meta-analysis. J Sci Med Sport.

[29] Wernbom M, Aagaard P (2020). Muscle fibre activation and fatigue with low-load blood flow restricted resistance exercise: an integrative physiology review. Acta Physiol.

[30] Centner C, Lauber B (2020). A systematic review and meta-analysis on neural adaptations following blood flow restriction training: what we know and what we don't know. Front Physiol.

[31] Zając A, Chalimoniuk M, Maszczyk A, Gołaś A, Lngfort J (2015). Central and peripheral fatigue during resistance exercise: a critical review. J Hum Kinet.

[32] Cook SB, Scott BR, Hayes KL, Murphy BG (2018). Neuromuscular adaptations to low-load blood flow restricted resistance training. J Sports Sci Med.

[33] Kubo K, Komuro T, Ishiguro N, Tsunoda N, Sato Y, Ishii N (2006). Effects of low-load resistance training with vascular occlusion on the mechanical properties of muscle and tendon. J Appl Biomech.

[34] Centner C, Jerger S, Lauber B, Seynnes O, Friedrich T, Lolli D (2022). Low-load blood flow restriction and high-load resistance training induce comparable changes in patellar tendon properties. Med Sci Sports Exerc.

[35] Centner C, Lauber B, Seynnes OR, Jerger S, Sohnius T, Gollhofer A (2019). Low-load blood flow restriction training induces similar morphological and mechanical Achilles tendon adaptations compared with high-load resistance training. J Appl Physiol.

[36] Luebbers PE, Fry AC, Kriley LM, Butler MS (2014). The effects of a 7-week practical blood flow restriction program on well-trained collegiate athletes. J Strength Cond Res.

[37] Blazevich AJ, Horne S, Cannavan D, Coleman DR, Aagaard P (2008). Effect of contraction mode of slow-speed resistance training on the maximum rate of force development in the human quadriceps. Muscle Nerve.

[38] Suga T, Okita K, Takada S, Omokawa M, Kadoguchi T, Yokota T (2012). Effect of multiple set on intramuscular metabolic stress during low-intensity resistance exercise with blood flow restriction. Eur J Appl Physiol.

[39] Cumming KT, Paulsen G, Wernbom M, Ugelstad I, Raastad T (2014). Acute response and subcellular movement of hsp27, alphab-crystallin and hsp70 in human skeletal muscle after blood-flow-restricted low-load resistance exercise. Acta Physiol.

[40] Madarame H, Ochi E, Tomioka Y, Nakazato K, Ishii N (2011). Blood flow-restricted training does not improve jump performance in untrained young men. Acta Physiol Hung.

[41] Abe T, Kawamoto K, Yasuda T, Kearns CF, Midorikawa T, Sato Y (2005). Eight days KAATSU-resistance training improved sprint but not jump performance in collegiate male track and field athletes. Int J KAATSU Training Res.

[42] Nielsen JL, Frandsen U, Prokhorova T, Bech RD, Nygaard T, Suetta C (2017). Delayed effect of blood-flow-restricted resistance training on rapid force capacity. Med Sci Sports Exerc.

[43] Behringer M, Behlau D, Montag JCK, McCourt ML, Mester J (2017). Low-intensity sprint training with blood flow restriction improves 100-m dash. J Strength Cond Res.

[44] Sakuraba K, Ishikawa T (2009). Effect of isokinetic resistance training under a condition of restricted blood flow with pressure. J Orthop Sci.

[45] Wilk M, Krzysztofik M, Filip A, Szkudlarek A, Lockie RG, Zajac A (2020). Does post-activation performance enhancement occur during the bench press exercise under blood flow restriction?. Int J Environ Res Public Health.

[46] Wilk M, Krzysztofik M, Filip A, Zajac A, Bogdanis GC, Lockie RG (2022). Short-term blood flow restriction increases power output and bar velocity during the bench press. J Strength Cond Res.

[47] Coffey VG, Hawley JA (2017). Concurrent exercise training: do opposites distract?. J Physiol.

[48] Schoenfeld BJ. Science and development of muscle hypertrophy. Champaign: Human Kinetics; 2016.

[49] Joseph AM, Pilegaard H, Litvintsev A, Leick L, Hood DA (2006). Control of gene expression and mitochondrial biogenesis in the muscular adaptation to endurance exercise. Essays Biochem.

[50] Taylor CW, Ingham SA, Ferguson RA (2016). Acute and chronic effect of sprint interval training combined with postexercise blood-flow restriction in trained individuals. Exp Physiol.

[51] Fahs CA, Rossow LM, Thiebaud RS, Loenneke JP, Kim D, Abe T (2014). Vascular adaptations to low-load resistance training with and without blood flow restriction. Eur J Appl Physiol.

[52] Geng Y, Wu X, Zhang L (2021). Effects of blood flow restriction training on blood perfusion and work ability of muscles in elite para-alpine skiers. Med Sci Sports Exerc.

[53] Pearson SJ, Hussain SR (2015). A review on the mechanisms of blood-flow restriction resistance training-induced muscle hypertrophy. Sports Med.

[54] Bodine SC, Stitt TN, Gonzalez M, Kline WO, Stover GL, Bauerlein R (2001). Akt/mtor pathway is a crucial regulator of skeletal muscle hypertrophy and can prevent muscle atrophy in vivo. Nat Cell Biol.

[55] Gundermann DM, Walker DK, Reidy PT, Borack MS, Dickinson JM, Volpi E (2014). Activation of mtorc1 signaling and protein synthesis in human muscle following blood flow restriction exercise is inhibited by rapamycin. Am J Physiol Endocrinol Metab.

[56] Figueiredo VC, Caldow MK, Massie V, Markworth JF, Cameron-Smith D, Blazevich AJ (2015). Ribosome biogenesis adaptation in resistance training-induced human skeletal muscle hypertrophy. Am J Physiol Endocrinol Metab.

[57] Kim H-G, Guo B, Nader GA (2019). Regulation of ribosome biogenesis during skeletal muscle hypertrophy. Exerc Sport Sci Rev.

[58] Brook MS, Wilkinson DJ, Mitchell WK, Lund JL, Phillips BE, Szewczyk NJ (2017). A novel d(2)o tracer method to quantify rna turnover as a biomarker of de novo ribosomal biogenesis, in vitro, in animal models, and in human skeletal muscle. Am J Physiol Endocrinol Metab.

[59] Sieljacks P, Wang J, Groennebaek T, Rindom E, Jakobsgaard JE, Herskind J (2019). Six weeks of low-load blood flow restricted and high-load resistance exercise training produce similar increases in cumulative myofibrillar protein synthesis and ribosomal biogenesis in healthy males. Front Physiol.

[60] Zak R, Rabinowitz M, Platt C (1967). Ribonucleic acids associated with myofibrils. Biochemistry.

[61] Stec MJ, Kelly NA, Many GM, Windham ST, Tuggle SC, Bamman MM (2016). Ribosome biogenesis may augment resistance training-induced myofiber hypertrophy and is required for myotube growth in vitro. Am J Physiol Endocrinol Metab.

[62] Nader GA, McLoughlin TJ, Esser KA (2005). Mtor function in skeletal muscle hypertrophy: increased ribosomal rna via cell cycle regulators. Am J Physiol Cell Physiol.

[63] Nielsen JL, Aagaard P, Prokhorova TA, Nygaard T, Bech RD, Suetta C (2017). Blood-flow restricted training leads to myocelullar macrophage infiltration and upregulation of heat-shock proteins, but no apparent muscle damage. J Physiol.

[64] Bjørnsen T, Wernbom M, Paulsen G, Berntsen S, Brankovic R, Stålesen H (2021). Frequent blood flow restricted training not to failure and to failure induces similar gains in myonuclei and muscle mass. Scand J Med Sci Sport.

[65] Paulsen G, Lauritzen F, Bayer ML, Kalhovde JM, Ugelstad I, Owe SG (1985). Subcellular movement and expression of hsp27, alphab-crystallin, and hsp70 after two bouts of eccentric exercise in humans. J Appl Physiol.

[66] Petrella JK, Kim J-S, Mayhew DL, Cross JM, Bamman MM (2008). Potent myofiber hypertrophy during resistance training in humans is associated with satellite cell-mediated myonuclear addition: a cluster analysis. J Appl Physiol.

[67] Bjørnsen T, Wernbom M, Paulsen G, Markworth JF, Berntsen S, D’Souza RF (2021). High-frequency blood flow-restricted resistance exercise results in acute and prolonged cellular stress more pronounced in type i than in type ii fibers. J Appl Physiol.

[68] Tatsumi R (2010). Mechano-biology of skeletal muscle hypertrophy and regeneration: possible mechanism of stretch-induced activation of resident myogenic stem cells. Anim Sci J.

[69] Larkin KA, Macneil RG, Dirain M, Sandesara B, Manini TM, Buford TW (2012). Blood flow restriction enhances post-resistance exercise angiogenic gene expression. Med Sci Sports Exerc.

[70] Allen RE, Sheehan SM, Taylor RG, Kendall TL, Rice GM (1995). Hepatocyte growth factor activates quiescent skeletal muscle satellite cells in vitro. J Cell Physiol.

[71] Aagaard P (2013). Hyperactivation of skeletal muscle stem cells with blood flow restricted resistance exercise: implications for muscle hypertrophy in sports and the clinical setting. J Sci Med Sport.

[72] Laurentino GC, Ugrinowitsch C, Roschel H, Aoki MS, Soares AG, Neves M (2012). Strength training with blood flow restriction diminishes myostatin gene expression. Med Sci Sports Exerc.

[73] Hulmi JJ, Ahtiainen JP, Kaasalainen T, Pollanen E, Hakkinen K, Alen M (2007). Postexercise myostatin and activin iib mrna levels: effects of strength training. Med Sci Sports Exerc.

[74] Park S, Kim JK, Choi HM, Kim HG, Beekley MD, Nho H (2010). Increase in maximal oxygen uptake following 2-week walk training with blood flow occlusion in athletes. Eur J Appl Physiol.

[75] Egan B, Zierath JR (2013). Exercise metabolism and the molecular regulation of skeletal muscle adaptation. Cell Metab.

[76] Hudlicka O, Brown MD (2009). Adaptation of skeletal muscle microvasculature to increased or decreased blood flow: role of shear stress, nitric oxide and vascular endothelial growth factor. J Vasc Res.

[77] Conceição MS, Chacon-Mikahil MP, Telles GD, Libardi CA, Junior EM, Vechin FC (2016). Attenuated pgc-1alpha isoforms following endurance exercise with blood flow restriction. Med Sci Sports Exerc.

[78] Christiansen D, Murphy RM, Bangsbo J, Stathis CG, Bishop DJ (2018). Increased fxyd1 and PGC-1α mrna after blood flow-restricted running is related to fibre type-specific ampk signalling and oxidative stress in human muscle. Acta Physiol.

[79] Egan B, Carson BP, Garcia-Roves PM, Chibalin AV, Sarsfield FM, Barron N (2010). Exercise intensity-dependent regulation of peroxisome proliferator-activated receptor coactivator-1 mrna abundance is associated with differential activation of upstream signalling kinases in human skeletal muscle. J Physiol.

[80] Irrcher I, Ljubicic V, Hood DA (2009). Interactions between ros and amp kinase activity in the regulation of pgc-1alpha transcription in skeletal muscle cells. Am J Physiol Cell Physiol.

[81] Groennebaek T, Jespersen NR, Jakobsgaard JE, Sieljacks P, Wang J, Rindom E (2018). Skeletal muscle mitochondrial protein synthesis and respiration increase with low-load blood flow restricted as well as high-load resistance training. Front Physiol.

[82] Lander EDC, Naess TC, Moen M, Cumming KT, Roberts LA, Raastad T. The dual ability of low-load blood flow restricted exercise: similar muscle growth but augmented increase in mitochondrial proteins compared to high-load resistance exercise. European Congress of Sports Science; Virtual Congress, 8–10 September, 2021.

[83] Drummond MJ, Fujita S, Abe T, Dreyer HC, Volpi E, Rasmussen BB (2008). Human muscle gene expression following resistance exercise and blood flow restriction. Med Sci Sports Exerc.

[84] Horiuchi M, Okita K (2012). Blood flow restricted exercise and vascular function. Int J Vasc Med.

[85] Kacin A, Strazar K (2011). Frequent low-load ischemic resistance exercise to failure enhances muscle oxygen delivery and endurance capacity. Scand J Med Sci Sports.

[86] Holloway TM, Snijders T, van Kranenburg J, van Loon LJ, Verdijk LB (2017). Temporal response of angiogenesis and hypertrophy to resistance training in young men. Med Sci Sports Exerc.

[87] Nielsen JL, Frandsen U, Jensen KY, Prokhorova TA, Dalgaard LB, Bech RD (2020). Skeletal muscle microvascular changes in response to short-term blood flow restricted training-exercise-induced adaptations and signs of perivascular stress. Front Physiol.

[88] Sundberg CJ (1994). Exercise and training during graded leg ischaemia in healthy man with special reference to effects on skeletal muscle. Acta Physiol Scand Suppl.

[89] Yasuda T, Brechue WF, Fujita T, Sato Y, Abe T (2008). Muscle activation during low-intensity muscle contractions with varying levels of external limb compression. J Sports Sci Med.

[90] Krustrup P, Soderlund K, Relu MU, Ferguson RA, Bangsbo J (2009). Heterogeneous recruitment of quadriceps muscle portions and fibre types during moderate intensity knee-extensor exercise: effect of thigh occlusion. Scand J Med Sci Sports.

[91] Jakobsgaard JE, Christiansen M, Sieljacks P, Wang J, Groennebaek T, de Paoli F (2018). Impact of blood flow-restricted bodyweight exercise on skeletal muscle adaptations. Clin Physiol Funct Imaging.

[92] Tabata S, Suzuki Y, Azuma K, Matsumoto H (2016). Rhabdomyolysis after performing blood flow restriction training: a case report. J Strength Cond Res.

[93] Clark BC, Manini TM (2017). Can KAATSU exercise cause rhabdomyolysis?. Clin J Sport Med.

[94] Bemben DA, Sherk VD, Buchanan SR, Kim S, Sherk K, Bemben MG (2022). Acute and chronic bone marker and endocrine responses to resistance exercise with and without blood flow restriction in young men. Front Physiol.

[95] Yasuda T, Ogasawara R, Sakamaki M, Ozaki H, Sato Y, Abe T (2011). Combined effects of low-intensity blood flow restriction training and high-intensity resistance training on muscle strength and size. Eur J Appl Physiol.

[96] Amani-Shalamzari S, Farhani F, Rajabi H, Abbasi A, Sarikhani A, Paton C (2019). Blood flow restriction during futsal training increases muscle activation and strength. Front Physiol.

[97] Amani-Shalamzari S, Sarikhani A, Paton C, Rajabi H, Bayati M, Nikolaidis PT (2020). Occlusion training during specific futsal training improves aspects of physiological and physical performance. J Sports Sci Med.

[98] Hosseini Kakhak SA, Kianigul M, Haghighi AH, Nooghabi MJ, Scott BR (2022). Performing soccer-specific training with blood flow restriction enhances physical capacities in youth soccer players. J Strength Cond Res.

[99] Sieljacks P, Matzon A, Wernbom M, Ringgaard S, Vissing K, Overgaard K (2016). Muscle damage and repeated bout effect following blood flow restricted exercise. Eur J Appl Physiol.

[100] Nosaka K, Clarkson PM (1995). Muscle damage following repeated bouts of high force eccentric exercise. Med Sci Sports Exerc.

[101] Husmann F, Mittlmeier T, Bruhn S, Zschorlich V, Behrens M (2017). Impact of blood flow restriction exercise on muscle fatigue development and recovery. Med Sci Sports Exerc.

[102] Loenneke JP, Thiebaud RS, Fahs CA, Rossow LM, Abe T, Bemben MG (2013). Blood flow restriction does not result in prolonged decrements in torque. Eur J Appl Physiol.

[103] Fujita T, Brechue WF, Kurita K, Sato Y, Abe T (2008). Increased muscle volume and strength following six days of low-intensity resistance training with restricted muscle blood flow. Int J KAATSU Train Res.

[104] Abe T, Yasuda T, Midorikawa T, Sato Y, Kearns CF, Inoue K (2005). Skeletal muscle size and circulating igf-1 are increased after two weeks of twice daily 'KAATSU' resistance training. Int J KAATSU Training Res.

[105] NSCA, French D, Ronda LT. Nsca's essentials of sport science. Champaign: Human Kinetics; 2021.

[106] Williams TD, Tolusso DV, Fedewa MV, Esco MR (2017). Comparison of periodized and non-periodized resistance training on maximal strength: a meta-analysis. Sports Med.

[107] Loenneke JP, Thiebaud RS, Abe T (2014). Does blood flow restriction result in skeletal muscle damage? A critical review of available evidence. Scand J Med Sci Sports.

[108] Sudo M, Ando S, Poole DC, Kano Y (2015). Blood flow restriction prevents muscle damage but not protein synthesis signaling following eccentric contractions. Physiol Rep.

[109] Drew MK, Finch CF (2016). The relationship between training load and injury, illness and soreness: a systematic and literature review. Sports Med.

[110] Wernbom M, Augustsson J, Thomee R (2006). Effects of vascular occlusion on muscular endurance in dynamic knee extension exercise at different submaximal loads. J Strength Cond Res.

[111] Fahs CA, Loenneke JP, Thiebaud RS, Rossow LM, Kim D, Abe T (2015). Muscular adaptations to fatiguing exercise with and without blood flow restriction. Clin Physiol Funct Imaging.

[112] Dirks ML, Wall BT, van de Valk B, Holloway TM, Holloway GP, Chabowski A (2016). One week of bed rest leads to substantial muscle atrophy and induces whole-body insulin resistance in the absence of skeletal muscle lipid accumulation. Diabetes.

[113] Loenneke JP, Abe T, Wilson JM, Thiebaud RS, Fahs CA, Rossow LM (2012). Blood flow restriction: an evidence based progressive model (review). Acta Physiol Hung.

[114] Takarada Y, Takazawa H, Ishii N (2000). Applications of vascular occlusion diminish disuse atrophy of knee extensor muscles. Med Sci Sports Exerc.

[115] Kubota A, Sakuraba K, Koh S, Ogura Y, Tamura Y (2011). Blood flow restriction by low compressive force prevents disuse muscular weakness. J Sci Med Sport.

[116] Hewitt JD, Harrelson JM, Dailiana Z, Guilak F, Fink C (2005). The effect of intermittent pneumatic compression on fracture healing. J Orthop Trauma.

[117] Loenneke JP, Young KC, Fahs CA, Rossow LM, Bemben DA, Bemben MG (2012). Blood flow restriction: rationale for improving bone. Med Hypotheses.

[118] Loenneke JP, Young KC, Wilson JM, Andersen JC (2013). Rehabilitation of an osteochondral fracture using blood flow restricted exercise: a case review. J Bodyw Mov Ther.

[119] Di Lemme S, Sanderson J, Celebrini RG, Dover GC (2020). A comprehensive nonoperative rehabilitation program including blood flow restriction for a talus fracture in a professional hockey player: a case report. Int J Athl Ther Train.

[120] Diwu W, Hu G, Zhou M, Bi L, Yan M, Wei H (2022). Effects of different intensities of intermittent pneumatic soft-tissue compression on bone defect repair. BMC Musculoskelet Disord.

[121] Holcomb WR (2006). Effect of training with neuromuscular electrical stimulation on elbow flexion strength. J Sports Sci Med.

[122] Head P, Waldron M, Theis N, Patterson SD (2020). Acute neuromuscular electrical stimulation (nmes) with blood flow restriction: the effect of restriction pressures. J Sport Rehabil.

[123] Inagaki Y, Madarame H, Neya M, Ishii N (2011). Increase in serum growth hormone induced by electrical stimulation of muscle combined with blood flow restriction. Eur J Appl Physiol.

[124] Slysz JT, Boston M, King R, Pignanelli C, Power GA, Burr JF (2021). Blood flow restriction combined with electrical stimulation attenuates thigh muscle disuse atrophy. Med Sci Sports Exerc.

[125] Tanaka M, Morifuji T, Sugimoto K, Akasaka H, Fujimoto T, Yoshikawa M (2021). Effects of combined treatment with blood flow restriction and low current electrical stimulation on capillary regression in the soleus muscle of diabetic rats. J Appl Physiol.

[126] Ohta H, Kurosawa H, Ikeda H, Iwase Y, Satou N, Nakamura S (2003). Low-load resistance muscular training with moderate restriction of blood flow after anterior cruciate ligament reconstruction. Acta Orthop Scand.

[127] Abe T, Kearns CF, Fujita S, Sakamaki M, Sato Y, Brechue WF (2009). Skeletal muscle size and strength are increased following walk training with restricted leg muscle blood flow: implications for training duration and frequency. Int J KAATSU Train Res.

[128] Beekley MD, Sato Y, Abe T (2005). KAATSU-walk training increases serum bone-specific alkaline phosphatase in young men. Int J KAATSU Train Res.

[129] Abe T, Kearns CF, Sato Y (2006). Muscle size and strength are increased following walk training with restricted venous blood flow from the leg muscle, KAATSU-walk training. J Appl Physiol.

[130] Ozaki H, Sakamaki M, Yasuda T, Fujita S, Ogasawara R, Sugaya M (2011). Increases in thigh muscle volume and strength by walk training with leg blood flow reduction in older participants. J Gerontol A Biol Sci Med Sci.

[131] Hughes L, Grant I, Patterson SD (2021). Aerobic exercise with blood flow restriction causes local and systemic hypoalgesia and increases circulating opioid and endocannabinoid levels. J Appl Physiol.

[132] Korakakis V, Whiteley R, Giakas G (2018). Low load resistance training with blood flow restriction decreases anterior knee pain more than resistance training alone: a pilot randomised controlled trial. Phys Ther Sport.

[133] Bittar ST, Pfeiffer PS, Santos HH, Cirilo-Sousa MS (2018). Effects of blood flow restriction exercises on bone metabolism: a systematic review. Clin Physiol Funct Imaging.

[134] Lazarczuk SL, Maniar N, Opar DA, Duhig SJ, Shield A, Barrett RS (2022). Mechanical, material and morphological adaptations of healthy lower limb tendons to mechanical loading: a systematic review and meta-analysis. Sports Med.

[135] Mersmann F, Laube G, Marzilger R, Bohm S, Schroll A, Arampatzis A (2021). A functional high-load exercise intervention for the patellar tendon reduces tendon pain prevalence during a competitive season in adolescent handball players. Front Physiol.

[136] Hortobagyi T, Houmard JA, Stevenson JR, Fraser DD, Johns RA, Israel RG (1993). The effects of detraining on power athletes. Med Sci Sports Exerc.

[137] Kubo K, Ikebukuro T, Yata H, Tsunoda N, Kanehisa H (2010). Time course of changes in muscle and tendon properties during strength training and detraining. J Strength Cond Res.

[138] Nakajima T, Kurano M, Iida H, Takano H, Oonuma H, Morita T (2006). Use and safety of KAATSU training: results of a national survey. Int J KAATSU Train Res.

[139] Australian Institute of Sport. Blood flow restriction training guidelines. Canberra: Australian Sports Commision; 2021. https://www.ais.gov.au/position_statements/best_practice_content/blood-flow-restriction-training-guidelines.

[140] Manimmanakorn A, Hamlin MJ, Ross JJ, Taylor R, Manimmanakorn N (2013). Effects of low-load resistance training combined with blood flow restriction or hypoxia on muscle function and performance in netball athletes. J Sci Med Sport.

[141] Christiansen D, Eibye K, Hostrup M, Bangsbo J (2020). Training with blood flow restriction increases femoral artery diameter and thigh oxygen delivery during knee-extensor exercise in recreationally trained men. J Physiol.

[142] Mitchell EA, Martin NRW, Turner MC, Taylor CW, Ferguson RA (1985). The combined effect of sprint interval training and postexercise blood flow restriction on critical power, capillary growth, and mitochondrial proteins in trained cyclists. J Appl Physiol.

